# Genome sequence of *Coxiella burnetii* KZQ2 isolated from a clinical strain in the Republic of Korea

**DOI:** 10.1128/MRA.01317-22

**Published:** 2023-07-21

**Authors:** Du-Gyeong Han, Seong-Han Kim, Se-Mi Jeon

**Affiliations:** 1 Department of Bacterial Disease Research, Center for Infectious Disease Research, National Institute of Infectious Disease, Korea National Institute of Health, Korea Disease Control and Prevention Agency, Cheongju-si, Republic of Korea; University of Maryland School of Medicine, Baltimore, Maryland, USA

**Keywords:** *Coxiella burnetii*, Q fever, genomes, MST

## Abstract

The genome of *Coxiella burnetii* KZQ2, isolated from clinical patients in Korea, is 2.04 MB long. Multispacer types were ST77, and phylogenetic tree analysis showed that KZQ2 is closely related to the CbuK_Q154 chronic strain isolated from human endocarditis patients in the USA.

## ANNOUNCEMENT

*Coxiella burnetii* is an intracellular zoonotic pathogen and proliferates logarithmically in acidic and low oxygen partial pressure environments (1). This organism has a plasmid genome such as QpDG, QpDV, QpH1, and QpRS or plasmid-like chromosomally integrated sequence, but the specific function of these conserved sequences has not been identified clearly (2). Here, we report the whole genome of *C. burnetii* isolated from clinical patients in Korea.

*C. burnetii* KZQ2 was isolated from whole blood collected from patients with nonspecific febrile illness has been residing in Cheongju-si, South Korea (3). To culture the bacteria, we inoculated 100 µL cell culture into immune-depressed 4-week-old Balb/c mice. We were approved by Centers for Disease Control and Prevention Institutional Review Board (Approved No. 2014-04CON-04-P-A) and the Institutional Animal Care and Use Committee (Approved No. KCDC-022-20-2A). Two months postinfection, the spleens of the infected animals were harvested (4 - 6). The homogenized spleens were then cultured in ACCM-D(-glucose) broth and incubated at 37°C, with 5% CO_2_ and 2.5% O_2_, for 28 days for bacterial enrichment.

Subsequently, plasmid typing and multispacer sequence typing (MST), which refers to Multi Spacers Typing - *Coxiella Burnetii* (https://ifr48.timone.univ-mrs.fr/mst/coxiella_burnetii/strains.html), were performed (7) and confirmed that the plasmid types and MST types of KZ-Q2 were identical. The plasmid type was QpRS, and spacers types were ST77. Genomic DNA was extracted from enriched bacteria cultured in ACCM-D(-glucose) using the Genomic DNA Prep Kit (Qiagen, Germantown, MD). DNA concentration required for library construction for whole genome sequencing (WGS) was measured using a BioTek Epoch spectrometer (Agilent Technologies, Santa Clara, CA). Subsequently, DNA libraries were prepared using a TruSeq DNA Library LT kit (Illumina, San Diego, CA). Then, a quality check was conducted using a Qubit 4 fluorometer (Invitrogen, Singapore) and a 2100 Bioanalyzer (Agilent technologies). DNA passed quality check was sequenced using a Miseq Reagent Kit version 3 and a MiSeq instrument (Illumina, San Diego, CA) to acquire 2 × 300 bp paired-end reads. Through Trimmomatic-0.36 (Usadellab, Germany) and BBMap v35.82 (https://sourceforge.net/projects/bbmap/), raw data were trimmed to eliminate low quality reads and index. Trimmed reads were assembled using SPAdes 3.13.0 (CAB SPbU, Russia). For annotation with strain *C. burnetii* RSA493 genome data (Accession No.: GCF_000007765.2), the WG annotation pipeline (CJ Bioscience, South Korea) was used. Default parameters were used for all software. In the WGS analysis, 3,925,852 reads were obtained (total 976,272,196 bp), and values of genome coverage and *N*_50_ were shown as 443.07 and 55,562, respectively. Results showed that the KZQ2 genome is 2,043,239 bp long, with 2,015 coding sequences, 61 contigs, 3 rRNA genes, 42 tRNA genes, and a GC ratio of 42.44%.

**Fig 1:**
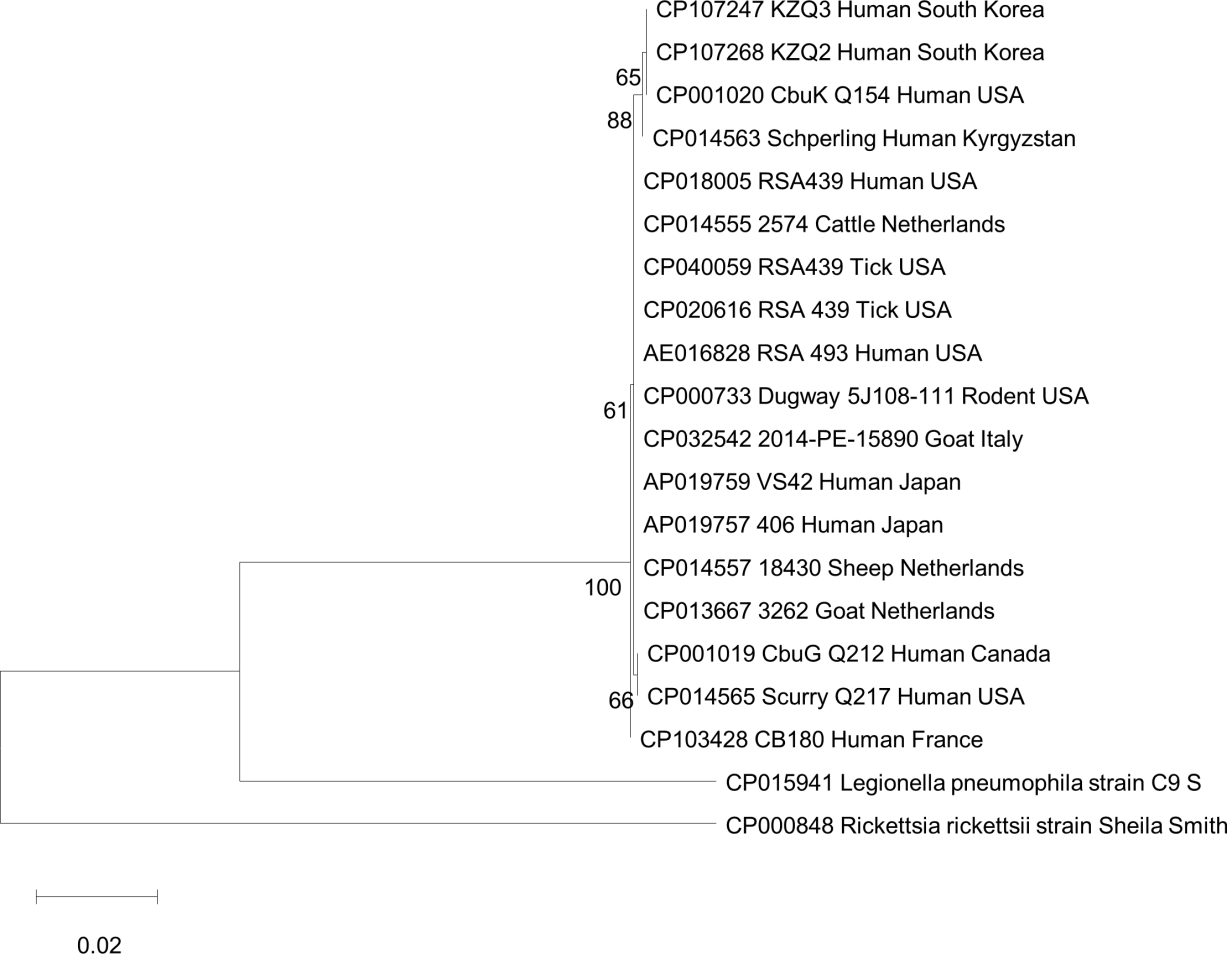
Phylogenetic analysis of the 16S rRNA gene sequences (1,466 bp) of isolate patients in South Korea and representative Coxiellaceae species. An unrooted phylogenetic tree was constructed with the maximum likelihood method and bootstrap values of 1,000 replicates using MEGA v.11.0.13 software.

The 16S rRNA sequences including 18 reference strains were aligned with BioEdit v7.7.1, and phylogenetic tree was constructed using the maximum likelihood method (8). Bootstrap analysis was performed with 1,000 pseudoreplicates. KZQ2 was observed to be closely related to CbuK_Q154, which harbors a QpRS plasmid and was isolated in a clinical patient with endocarditis (Figure 1).

## Data Availability

The whole genome shotgun sequencing project of *Coxiella burnetii* KZQ2 has been deposited at GenBank under the accession number JAFCIU010000000 and in the Sequence Read Archive accession number SRR22439205.
